# Impact of C-Terminal Chemistry on Self-Assembled Morphology of Guanosine Containing Nucleopeptides

**DOI:** 10.3390/molecules25235493

**Published:** 2020-11-24

**Authors:** Katherine Boback, Katherine Bacchi, Sarah O’Neill, Samantha Brown, Jovelt Dorsainvil, Jillian E. Smith-Carpenter

**Affiliations:** Department of Chemistry and Biochemistry, Fairfield University, 1073 N. Benson Rd, Fairfield, CT 06824, USA; kboback21@amherst.edu (K.B.); katherine.bacchi@student.fairfield.edu (K.B.); sarah.oneill@student.fairfield.edu (S.O.); samantha.brown2@student.fairfield.edu (S.B.); jovelt.dorsainvil@student.fairfield.edu (J.D.)

**Keywords:** nucleopeptide, self-assembly, guanosine, G-quartet

## Abstract

Herein, we report the design and characterization of guanosine-containing self-assembling nucleopeptides that form nanosheets and nanofibers. Through spectroscopy and microscopy analysis, we propose that the peptide component of the nucleopeptide drives the assembly into β-sheet structures with hydrogen-bonded guanosine forming additional secondary structures cooperatively within the peptide framework. Interestingly, the distinct supramolecular morphologies are driven not by metal cation responsiveness common to guanine-based materials, but by the C-terminal peptide chemistry. This work highlights the structural diversity of self-assembling nucleopeptides and will help advance the development of applications for these supramolecular guanosine-containing nucleopeptides.

## 1. Introduction

Synthetically and strategically designed self-assembly systems composed of amino acids have resulted in supramolecular structures capable of a vast range of functions, from serving as interactive platforms for reactions [1,2,3] to drug delivery applications [4,5]. As chemists and material scientists look to further diversify the structures and functions of supramolecular systems to better capture the complex and synergistic assemblies of biological systems, the modification of short peptides with other biomolecules has been one documented strategy. Self-assembling amyloid peptides modified by nucleic acids [6] or by lipid moieties [7,8] were some of the first systems studied to explore these chimeric molecules.

Nucleopeptides, molecules containing nucleic acid and amino acid building blocks, are particularly interesting as the amino acid component can facilitate assembly into supramolecular structures while the nucleic acid component confers added molecular recognition elements based on various hydrogen bonding motifs. The incorporation of the hemiprotonated C-C^+^ base pair, or *i*-motif, with an amyloid Aβ peptide derivative to yield soluble nanotubes was an early example of supramolecular cooperation between peptide and nucleic acid components in a self-assembling nucleopeptide [6]. Xu’s group then designed hydrogelating self-assembling nucleopeptides, with [9,10,11] and without additional sugar moieties [12]. The material properties of the resulting hydrogels could be modulated through sequence modification or by co-assembly of base-pairing nucleopeptides [9,12]. These nucleopeptides have since been used for various biomaterial applications, including the ability to sequester ATP in cellular conditions as a method to increase the efficacy of doxorubicin treatment in cancer cells [13]. Recently, Suggs et al. designed an expanded library of 16 nucleopeptides (base-XFF-OH, with X = Phe, Ala, Gly, or Lys) [14]. The hydrogelation and subsequent mechanical properties of the nucleopeptide assemblies were systematically controlled through nucleobase and amino acid composition and displayed low cytotoxicity against 3T3 fibroblast cells [14].

In addition to Watson-Crick-Franklin hydrogen bonding, nucleic acids, and guanine in particular, can facilitate molecular recognition along the Hoogsteen face of the base to form distinct structural motifs, G-quartets, and G-ribbons. G-quartets are a cyclic arrangement of four guanine residues that hydrogen bond along the Hoogsteen face and, in sequences of G-rich DNA, such as telomeric sequences [15], the G-quartets stack to form larger G-quadruplex structures. When guanosine is incorporated into self-assembling molecules, as with such examples as lipophilic moieties [16] and terpyridine tethered peptide scaffold [17], higher order G-quartet structures can also be formed when coordinated by metal cations, most commonly K^+^ or Na^+^. A second guanosine-based motif, the G-ribbon, can form an extended sheet of interacting guanines. It has been shown that lipophilic-derivatized guanosine molecules can form either G-quartet or G-ribbon based supramolecular structures based on the presence of metal cations [18,19].

**Figure 1:**
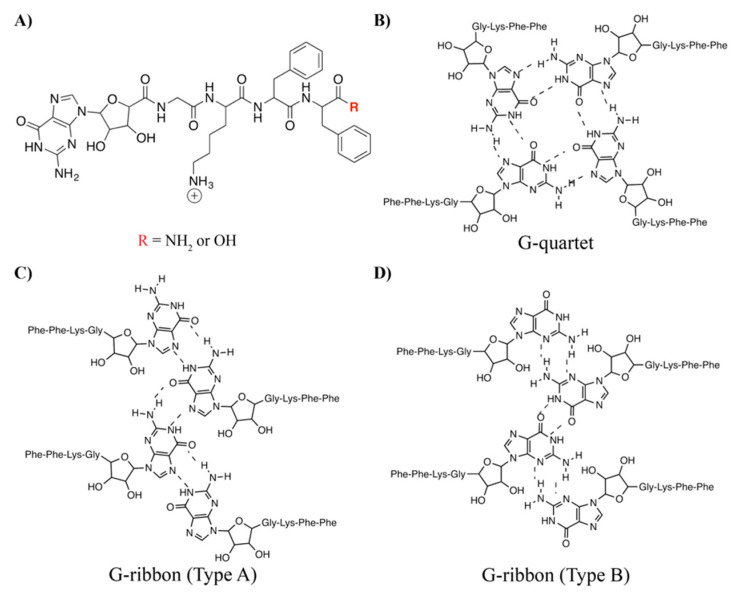
Structure of nucleopeptide and potential guanosine-based secondary structures. (**A**) Nucleopeptide synthesized by modifying the short peptide Gly-Lys-Phe-Phe with guanosine. Nucleopeptides with amide and with carboxylic acid C-terminal were studied. Proposed assembly structures utilizing guanosine based on either a G-quartet (**B**) or G-ribbon (**C**,**D**) architecture.

Inspired by previously reported nucleopeptide constructs [6,9,10,11,12,13,14], we synthesized and assembled a tetrapeptide (Gly-Lys-Phe-Phe) that is modified on the *N*-terminus with guanosine. We have referred to guanosine as gs in this work to differentiate between the guanine nucleobase used in previous nucleopeptide studies [9,12,14]. The Phe-Phe-dyad is a common hydrophobic core that facilitates higher order assembly in many short self-assembling peptide systems [20,21,22]. To the Phe-Phe core, we added a lysine residue to aid in solubility and a glycine residue to allow for more flexibility as the nucleopeptide transitions from peptide to nucleoside components (Figure 1A). The synthetic characterization for these nucleopeptides, one constructed with a carboxylic acid C-terminus and one constructed with an amidated C-terminus, are provided in Supplementary Materials (Figures S2–S5). We hypothesized that the nucleopeptides would form β-sheet rich supramolecular structures that also contain unique guanosine hydrogen-bonding motifs, namely G-quartets or G-ribbons (Figure 1B–D).

## 2. Results and Discussion

The gs-GKFF-OH nucleopeptide was assembled in 20% (*v*/*v*) acetonitrile in Millipore water to yield a 2 mg mL^−1^ final concentration (0.2 wt%) and pH 4.5, with some solutions containing 1 equivalent of KCl to coordinate potential G-quartet structures with K^+^ [23,24,25,26]. After 24 h, a loose gel, with only a portion of the sample remaining at the tube bottom during the vial inversion test, was visible (Figure S6A). However, after 48 h, a completely transparent gel formed and the entire sample remains in place during vial inversion. The transparent gel remains stable, with no nucleopeptide precipitation, for over one week (Figure S6B). The same assembly procedure was followed for the gs-GKFF-NH_2_ nucleopeptide. The 2 mg mL^−1^ assembly of the amide C-terminal construct stays in solution after 24 h (Figure S6C) and remains a solution even after weeks (data not shown). While the hydrogel formation of guanosine containing gs-GKFF-OH is consistent with some guanine nucleobase containing peptides with a FF dyad [9,12], this nucleopeptide differs from the guanine containing tripeptides (gua-KFF-OH and gua-GFF-OH) reported by Suggs et al. [14], as they did not form a hydrogel and no nanofibers were characterized.

**Figure 2:**
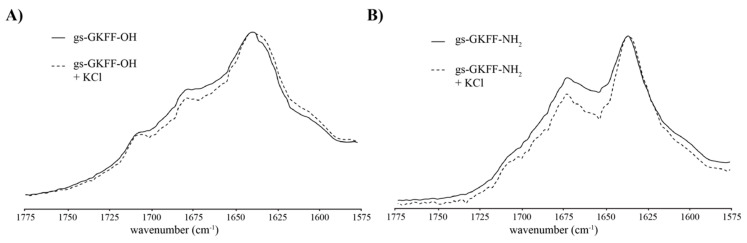
FTIR analysis in the absence of KCl (solid line) and presence of 1 eq. KCl (dashed line) for nucleopeptide assemblies of gs-GKFF-OH (**A**) and gs-GKFF-NH_2_ (**B**) after one week of assembly.

We investigated the higher order assembly molecular interactions of the guanosine containing nucleopeptides (Figure 1A) with Fourier transform infrared spectroscopy (FTIR) analysis of the amide I region (1775–1575 cm^−1^). In the zwitterionic carboxylic acid gs-GKFF-OH nucleopeptide, there is a distinct and intense peak at 1638 cm^−1^, common with β-sheet forming assemblies (Figure 2A) [27]. Additionally, the peak at 1678 cm^−1^ is consistent with the involvement of the C6 carbonyl of guanosine in a hydrogen-bond [28,29]. Since the small peak at 1707 cm^−1^ is still red-shifted compared to reported peaks of the non-hydrogen bonding C6 guanosine (ca. 1724 cm^−1^) [28,29], the peak is likely associated with a population of guanosine residues participating in a weaker hydrogen-bonding environment. The addition of KCl to the gs-GKFF-OH assemblies alters the shape of the peak centered at 1638 cm^−1^ (Figure 2A). The second derivative spectra of gs-GKFF-OH assemblies with and without additional KCl show unique negative peak positions that resolve a shoulder at 1636 cm^−1^ in assemblies without KCl and a shoulder at 1645 cm^−1^ in assemblies with additional KCl (Figure S7). The peak at 1645 cm^−1^ indicates a population of unordered nucleopeptide [27]. The peak at 1636 cm^−1^ suggests heterogeneity in the β-sheet structures in the nucleopeptide gs-GKFF-OH assemblies.

The FTIR of the amide C-terminus gs-GKFF-NH_2_ nucleopeptide assembly also show signals associated with β-sheet formation (1636 cm^−1^) and the hydrogen-bonded guanosine C6 (1674 cm^−1^) (Figure 2B). The second derivative spectra of the gs-GKFF-NH_2_ nucleopeptide assembled with or without additional KCl show nearly identical negative peak positions, indicating that KCl has little effect on the amide terminated nucleopeptide compared to the carboxylate terminated construct (Figure S7). While the FTIR data suggests that both nucleopeptide assemblies contain β-sheets and hydrogen bonded guanosines, differences in position and relative intensity, along with a considerably smaller shoulder present at 1707 cm^−1^ in the amide terminated nucleopeptide (Figure 2B), suggest different arrangements of the underlying secondary structures at the molecular level.

**Figure 3:**
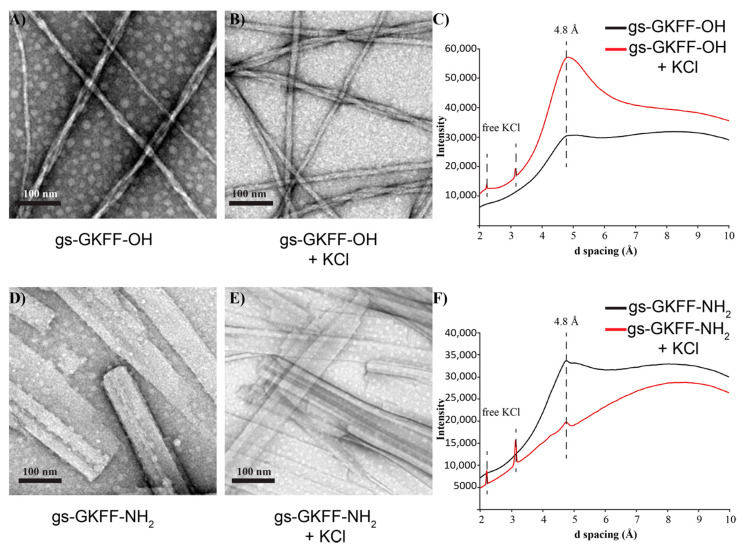
TEM images of gs-GKFF-OH after one week of assembly without additional KCl (**A**) and with 10 eq. KCl (**B**). TEM images of gs-GKFF-NH_2_ after one week of assembly without additional KCl (**D**) and with 10 eq. KCl (**E**). PXRD analysis shows d-spacing for β-sheet laminates at 4.8 Å (**C**,**F**).

These differences in molecular arrangements are further evidenced by the difference in supramolecular morphology as characterized by transmission electron microscopy (TEM). Short, twisting nanofibers are seen in the gs-GKFF-OH assemblies (Figure 3A,B). While the larger nanofiber has an average width of 15 nm, these nanofibers are composed of smaller, single fibers with an average width of 4 nm (Figure 3A,B and Figure S8). In TEM images of gs-GKFF-OH assembled without additional KCl, there is more heterogeneity in the bundling of individual fibers compared to assemblies with KCl that show more consistent bundling of fibers (Figure 3A,B). As the C-terminus is negatively charged under the assembly condition of pH 4.5, the K^+^ might serve to electrostatically counter the carboxylate group and facilitate a higher degree of bundling. The gs-GKFF-NH_2_ nucleopeptide assembles into flat nanosheets (Figure 3D,E). The assemblies were lyophilized and analyzed by powder X-ray diffraction (PXRD). The PXRD analysis shows a diffraction peak in both assemblies at 4.8 Å, which is the distance between two neighboring peptides in a β-sheet (Figure 3C,F). The broad peak from the d-spacing at 4.8 Å in the gs-GKFF-OH assemblies, compared to the sharper peaks in the gs-GKFF-NH_2_ assemblies, indicate more heterogeneity in the gs-GKFF-OH nucleopeptides (Figure 3C,F). The heterogeneity is clearly seen in the TEM images, as the gs-GKFF-OH nucleopeptide nanofibers bundle with varying helical twists (Figure 3A,B), while the gs-GKFF-NH_2_ nucleopeptides form more uniform nanosheets (Figure 3D,E) [30,31]. Additionally, the relative higher intensity of the PXRD signals for the gs-GKFF-OH assemblies in the presence of KCl is attributed to the bundling of the nanofibers that places a greater population of assembly sample in the same orientation (Figure 3C). Samples with components that are randomly arranged will have a lower intensity owing to multiple orientations [31]. The TEM and PXRD data combine to suggest that although the gs-GKFF-OH and gs-GKFF-NH_2_ nucleopeptides exhibit different morphologies, the assemblies are similarly composed of β-sheet interactions.

The absence of PXRD reflections for stacked G-quartets (Figure 3C,F), which is typically around 3.3 Å [17,28], with the presence of hydrogen-bonded guanosine in the FTIR analysis (Figure 2), suggests that the supramolecular morphologies of these nucleopeptides are dictated by the peptide component. Specifically, it is the molecular interactions of the C-terminus that play an important role in directing the supramolecular morphology [32]. This is further supported by analysis of nucleopeptides assembled without the presence of KCl to coordinate guanosines into G-quartets. As both nucleopeptide assemblies displayed similar FTIR, TEM, and XRD analysis when assembled without KCl (Figure 2 and Figure 3), this indicates that the typical metal cation responsiveness of guanosine seen in other guanine-based assembles is not present in these nucleopeptides [19,28,33].

In the gs-GKFF-OH nucleopeptide assembly, the carboxylate C-terminus would be negatively charged at pH 4.5. In an effort to delocalize these charges as the nucleopeptide grows into higher-order structures, we propose that the electrostatic repulsion forces neighboring nucleopeptides to shift slightly along the fibers’ long axis into the twisting, small nanofibers seen in the TEM images (Figure 3A,B). This assembly would then position the more hydrophobic guanosines in the interior of the nanofiber in proximity to form single hydrogen bond stabilized G-quartets (Figure 2A). In contrast, we propose the flat sheets formed by the gs-GKFF-NH_2_ nucleopeptide better accommodate the planar hydrogen bonded network of guanosines in a G-ribbon structure. The amide terminus, as it contains both hydrogen bond donor and accepting functionalities, could then stabilize interactions between neighboring nucleopeptides to produce the wide sheets seen in the TEM images (Figure 3).

## 3. Materials and Methods

### 3.1. Materials

The following reagents were purchased from commercial vendors (Fisher Scientific (Waltham, MA, USA) or Sigma Aldrich (St. Louis, MO, USA)) and used without further purification: guanosine (Fisher Scientific), p-toluenesulfonic acid (Fisher Scientific), 2,2-dimethoxypropane (Fisher Scientific), acetone, (Fisher Scientific), 2,2,6,6,-tetramethyl-1-piperidinyloxyl (Fisher Scientific), [bis(acetoxy-iodo]benzene (Fisher Scientific), dimethylformamide (Fisher Scientific), Fmoc-Phe wang resin (Fisher Scientific), *N*,*N*,*N′*,*N′*-tetremethyl-*O*-(1*H*-benzotriazol-1-yl)uranium hexafluorophosphate (HBTU) (TCI America, Tokyo, Japan), fluorenylmethyloxycarbonyl (Fmoc) protected amino acids (Chem-Impex Int., Inc., Wood Dale, IL, USA), *N*-methylmorpholine (Fisher Scientific), dichloromethane (Fisher Scientific), *N*,*N′*-diisopropylcarbodiimide (DIC) (Fisher Scientific), ethyl cyano(hydroxylmino)acetate (Oxyma Pure) (Sigma Aldrich), trifluoroacetic acid (TFA) (Fisher Scientific), triisopropylsilane (TIPS) (Fisher Scientific), diethyl ether (Fisher Scientific), acetonitrile (Fisher Scientific), alpha-cyano-4-hydroxycinnamic acid (CHCA) (Ricca Chemical, Arlington, TX, USA), 4% uranyl acetate solution (Electron Microscopy Sciences). All water used to prepare assembly solutions was 18.2 MΩ ultrapurified water.

### 3.2. Synthesis of 2′,3′-O-Isopropylideneguanosine-5′-Carboxylic Acid

The *O*-diol-protected guanosine acid derivative was prepared based on previous reports [34]. Briefly, a solution is prepared by adding 1.5 g of guanosine and 1.05 g (1 equivalent) of p-toluenesulfonic acid to 60 mL of acetone. While stirring at room temperature, 15 mL (2.5 equivalents) of 2,2-dimethoxypropane is added and stirring is continued overnight. After the solvent is removed under vacuum, 10 mL of water and 1 equivalent of NaHCO_3_ is added. The solution is stirred for two hours and then an additional 10 mL of saturated NaHCO_3_ is added. After 2 h, the 2′,3′-isopropylidene-protected nucleoside is filtered and washed with water. To form the carboxylic acid derivative, combine catalytic amounts of 2,2,6,6-tetramethyl-1-piperidinyloxyl (TEMPO) (0.5 equivalent), [bis(acetoxy)-iodo]benzene (BAIB) (2.2 equivalents) and the 2′,3′-isopropylidene-protected nucleoside (1 equivalent) in a 1:1 acetonitrile:water solution with NaHCO_3_ (2 equivalents). Stir reaction for 3 h and then filter to yield 2′,3′-*O*-isopropylideneguanosine-5′-carboxylic acid. Wash filtrate first with diethyl ether and then acetone. ^1^H NMR: (400 MHz, DMSO-*d*_6_): δ 1.3 (m, 3H), 1.5 (m, 3H), 4.3 (s, 1H), 5.0 (m, 2H), 5.9 (t, 1H), 6.8 (s, 2H), 8.4 (s, 1H), 11.5 (s, 1H) (Figure S1).

### 3.3. Synthesis of Nucleopeptide

The peptide component of the nucleopeptide was synthesized on PS3 synthesizer (Gyros Protein Technologies, Tucson, AZ, USA) on wang resin at 0.1 mmol scale, using the manufacturer’s standard coupling and wash times for Fmoc solid-phase peptide synthesis. After the N-terminal glycine was added, the final Fmoc was removed, the resin was washed with DMF and DCM, and transferred to a fritted syringe (Torviq, Tucson, AZ, USA). The coupling of the 2′,3′-*O*-isopropylideneguanosine-5′-carboxylic acid (3 equivalents) to the free amine *N*-terminus with Oxyma Pure (3 equivalents) and DIC (3 equivalents) in 5 mL of DMF. After two hours, the resin was washed with DMF and DCM.

The modified peptide was deprotected and cleaved from the resin with a 5 mL TFA/TIPS/H_2_O (95%/2.5%/2.5%) cleavage cocktail for 2 h at room temperature, while in the fritted syringe. The peptide-containing solution was eluted from the fritted syringe into cold diethyl ether (25 mL) and the precipitate was centrifuged at 1300× *g* for 10 min. The ether was decanted, the crude peptide pellet was resuspended in an additional 25 mL of cold ether, and then centrifuged a second time. The ether was decanted and the crude peptide pellet was dried in vacuo to remove the remaining ether. Then the dried peptide pellet was solubilized in a minimum volume of 10% acetonitrile and filtered through a 22 mm syringe filter into an HPLC vial. The peptide was HPLC (Shimadzu, Tokyo, Japan) purified using C-18 semi-preparative column (XBridge BEH, Waters, Milford, MA, USA) with a flow rate of 3 mL/min over a linear gradient of 10–30% ACN in 20 min. Fractions containing the pure peptide were confirmed by MALDI-TOF (Shimadzu, Tokyo, Japan), then combined and lyophilized (Figures S2 and S4). ^1^H NMR (JEOL) was used to characterize the final purified nucleopeptide in DMSO-*d*_6_. gsGKFF-OH: (400 MHz, DMSO-*d*_6_): δ 1.2 (s, 2H), 1.5 (m, 4H), 2.7 (m, 3H), 3.0 (m, 3H), 3.7 (dd, 1H), 3.9 (dd, 1H), 4.2 (s, 1H), 4.3 (m, 2H), 4.4 (m, 1H), 4.5 (m, 2H), 5.6 (d, 1H), 5.7 (s, 1H), 5.9 (d, 1H), 6.5 (s, 2H), 7.2 (m, 10H), 7.7 (s, 2H), 8.0 (t, 2H), 8.1 (s, 2H), 8.3(d, 1H), 10.9 (s, H1) (Figure S3). gsGKFF-NH_2_: (400 MHz, DMSO-*d*_6_): δ 1.2 (s, 2H), 1.5 (m, 4H), 2.7 (m, 3H), 3.0 (m, 3H), 3.7 (dd, 1H), 3.9 (dd, 1H), 4.2 (s, 1H), 4.3 (m, 2H), 4.5 (m, 3H), 5.7 (bd, 2H), 5.9 (d, 1H), 6.5 (s, 2H), 7.2 (m, 11H), 7.8 (s, 2H), 8.1 (d, 1H), 8.2 (t, 2H), 8.3(m, 2H), 10.8 (s, H1) (Figure S5).

### 3.4. Assembly Preparation

All nucleopeptide assemblies were prepared by dissolving lyophilized nucleopeptide powder with a 20% acetonitrile in water solution (% vol) to a final concentration of 2 mg/mL. The peptide assembly mixture was vortexed and sonicated with heat for thirty minutes to ensure that all nucleopeptide went into solution. To some solutions, potassium chloride (1 equivalent) was added prior to sonication. The assembly solutions were allowed to assemble at room temperature in capped microcentrifuge tubes. The pH of the assembly solutions was determined to by pH 4.5 by dropping several (~20 μL) microliters of solution onto litmus paper.

### 3.5. Fourier Transform Infrared Spectroscopy

An 8 μL aliquot of the assembly was added to the ATR diamond crystal (Alpha II, Bruker, Rheinstetten, Germany) and allowed to dry to forma thin film. After the water peak disappeared, the IR was acquired from 1500–1800 cm^−1^ (50 scans with a 2 cm^−1^ resolution).

### 3.6. TEM Imaging

Carbon supported 200 mesh copper grids (Electron Microscopy Sciences) were used for all TEM. A 10 μL aliquot of the assembly mixture was pipetted onto the grid and allowed to sit for approximately 1–2 min. The excess solution was wicked away using a straight edge of a piece of filter paper. Then 10 μL of a 2% uranyl acetate solution was added to the carbon grid and allowed to sit for another 1–2 min. The excess stain was then wicked away and the grids were stored in vacuo until imaging. TEM images were taken at magnifications from 9300× to 23,000× using a tungsten filament with an accelerating voltage of 120 kV (FEI Tecnai T12, Hillsboro, OR, USA).

### 3.7. Powder X-ray Diffraction

Room temperature diffraction data were collected with a Rigaku MicroMax-007HF source (Cu Kα; λ = 1.54178 Å) coupled to a Saturn994+ CCD detector. Line profiles of the 2D diffraction data were integrated and processed with the Rigaku 2DP software package (Rigaku, Tokyo, Japan).

## 4. Conclusions

While the β-sheet forming peptide component prevents the guanosine from forming π-π stacking interactions (Figure 3C,F), the FTIR analysis shows that the guanosine moieties hydrogen bond (Figure 2). We propose that the gs-GKFF-OH nucleopeptide is able to accommodate individual G-quartets within the peptide framework. The incorporation of G-quartet structures into supramolecular assemblies opens the possibility for nucleopeptides to be supramolecular catalysts. As other recent examples of guanine-based supramolecular structures have been shown to catalyze peroxidase reactions in the presence of hemin [35] and have provided a chiral scaffold for enantiomeric Freidel-Crafts alkylation [36], guanosine containing nucleopeptides should also be tested for emergent catalytic properties. Additionally, as the mutualism between nucleic acids and peptides in biological systems has positioned nucleopeptides [37] and co-assemblies of peptides and nucleic acids [38,39] as important systems in understanding chemical evolution, the self-assembled guanosine containing nucleopeptides reported in this study may inform future studies of dynamic chemical networks.

In conclusion, we have designed and characterized two new self-assembling nucleopeptides that contain both β-sheet interactions and hydrogen-bonding guanosines (Figure 2). We propose, that while the peptide component acts as the scaffold for the higher order assembly, the flexibility of guanine to form either G-quartets or G-ribbons acts cooperatively with the peptide scaffold to yield distinct supramolecular morphologies depending on the C-terminus peptide chemistry (Figure 3). As continued investigations are currently underway to elucidate further molecular level details of these assemblies, we believe that the characterization of these nucleopeptides presented in this work will help advance functional applications of nucleopeptides.

## Supplementary Materials

The Supplementary Materials are available online. Characterization data (HPLC, MALDI-TOF MS, ^1^H NMR) for the nucleopeptides, second derivative FTIR analysis, and vial inversion test.

## References

[1] Chatterjee A., Afrose S.P., Ahmed S., Venugopal A., Das D. (2020). Cross-β amyloid nanotubes for hydrolase-peroxidase cascade reactions. Chem. Commun..

[2] Duncan K.L., Ulijn R.V. (2015). Short peptides in minimalistic biocatalyst design. Biocatalysis.

[3] Makhlynets O.V., Gosavi P.M., Korendovych I.V. (2016). Short self-assembling peptides are able to bind to copper and activate oxygen. Angew. Chem. Int. Ed..

[4] Nagai Y., Unsworth L.D., Koutsopoulos S., Zhang S. (2006). Slow release of molecules in self-assembling peptide nanofiber scaffold. J. Control Release.

[5] Altunbas A., Lee S.J., Rajasekaran S.A., Schneider J.P., Pochan D.J. (2011). Encapsulation of curcumin in self-assembling peptide hydrogels as injectable drug delivery vehicles. Biomaterials.

[6] Liu P., Ni R., Mehta A.K., Childers W.S., Lakdawala A., Pingali S.V., Thiyagarajan P., Lynn D.G. (2008). Nucleobase-directed amyloid nanotube assembly. J. Am. Chem. Soc..

[7] Ni R., Childers W.S., Hardcastle K.I., Mehta A.K., Lynn D.G. (2012). Remodeling cross-β nanotube surfaces with peptide/lipid chimeras. Angew. Chem. Int. Ed..

[8] Hartgerink J.D., Beniash E., Stupp S.I. (2001). Self-assembly and mineralization of peptide-amphiphile nanofibers. Science.

[9] Li X., Kuang Y., Shi J., Gao Y., Lin H., Xu B. (2011). Multifunctional, biocompatible supramolecular hydrogelators consist only of nucleobase, amino acid, and glycoside. J. Am. Chem. Soc..

[10] Li X., Du X., Gao Y., Shi J., Kuang Y., Xu B. (2012). Supramolecular hydrogels formed by the conjugates of nucleobases, Arg-Gly-Asp (RGD) peptides, and glucosamine. Soft Matter.

[11] Wu D., Zhou J., Shi J., Du X., Xu B. (2014). A naphthalene-containing amino acid enables hydrogelation of a conjugate of nucleobase-saccharideamino acids. Chem. Commun..

[12] Li X., Kuang Y., Lin H., Gao Y., Shi J., Xu B. (2011). Supramolecular nanofibers and hydrogels of nucleopeptides. Angew. Chem. Int. Ed..

[13] Wuang H., Feng Z., Qin Y., Wang J., Xu B. (2018). Nucleopeptide assemblies selectively sequester ATP in cancer cells to increase the efficacy of doxorubicin. Angew. Chem. Int. Ed..

[14] Baek K., Noblett A.D., Ren P., Suggs L.J. (2019). Design and characterization of nucleopeptides for hydrogel self-assembly. ACS Appl. Bio Mater..

[15] Henderson E., Hardin C.C., Walk S.K., Tinoco I., Blackburn E.H. (1987). Telomeric DNA oligonucleotides form novel intramolecular structures containing guanine-guanine base pairs. Cell.

[16] Wang X., Zhou L., Wang H., Luo Q., Xu J., Liu J. (2011). Reversible organogels triggered by dynamic K+ binding and release. J. Colloid Interface Sci..

[17] Zhu X., Zou R., Sun P., Wang Q., Wu J. (2018). A supramolecular peptide polymer from hydrogen-bond and coordination-driven self-assembly. Polym. Chem..

[18] Gonnelli A., Pieraccini S., Baldassarri E.J., Funari S., Masiero S., Ortore M.G., Mariani P. (2020). Metallo-respnsive self-assembly of lipophilic guanines in hydrocarbon solvents: A systematic SAXS structural characterization. Nanoscale.

[19] Arnal-Herault C., Pasc A., Michau M., Cot D., Petit E., Barboiu M. (2007). Functional G-quartet macroscopic membrane films. Angew. Chem. Int. Ed..

[20] Görbitz C.H. (2001). Nanotube formation by hydrophobic dipeptides. Chem. Eur. J..

[21] Reches M., Gazit E. (2003). Casting metal nanowires within discrete self-assembled peptide nanotubes. Science.

[22] Mason T.O., Chirgadze D.Y., Levin A., Adler-Abramovich L., Gazit E., Knowles T.P., Buell A.K. (2014). Expanding the solvent chemical space for self-assembly of dipeptide nanostructures. ACS Nano.

[23] Davis J.T. (2004). G-quartets 40 years later: From 5′-GMP to molecular biology and supramolecular chemistry. Angew. Chem. Int. Ed..

[24] Gottarelli G., Spada G.P. (2004). The stepwise supramolecular organization of guanosine derivatives. Chem. Rec..

[25] Lena S., Masiero S., Pieraccini S., Spad G.P. (2009). Guanosine hydrogen-bonded scaffolds: A new way to control the bottom-up realization of well-defined nanoarchitectures. Chem. Eur. J..

[26] Dash J., Saha P. (2016). Functional architectures derived from guanine quartets. Org. Biomol. Chem..

[27] Barth A. (2007). Infrared spectroscopy of proteins. Biochim. Biophys. Acta Bioenerg..

[28] Xiao S., Davis J.T. (2018). G4-quartet hydrogels from 5′hydrazino-guanosine for the non-covalent and covalent remediation of contaminants from water. Faraday Discuss..

[29] Setnička V., Urbanová M., Volka K., Nampally S., Lehn J. (2006). Investigation of guanosine-quartet assemblies by vibrational and electronic circular dichroism spectroscopy, a novel approach for studying supramolecular entities. Chem. Eur. J..

[30] Singh V., Snigdha K., Singh C., Sinha N., Thakur A.K. (2015). Understanding the slef-assembly f Fmoc-phenylalanine to hydrogel formation. Soft Matter.

[31] Jian Z., Hejing W. (2003). The physical meanings of 5 basic parameters for an X-ray diffraction peak and their application. Chin. J. Geochem..

[32] Xie Y., Wang X., Huang R., Qi W., Wang Y., Su R., He Z. (2015). Electrostatic and aromatic interaction-directed supramolecular self-assembly of a designed Fmoc-tripeptide into helical nanoribbons. Langmuir.

[33] Meng L., Liu K., Mo S., Mao Y., Yi T. (2013). From G-quartets to G-ribbon gel by concentration and sonication control. Org. Biomol. Chem..

[34] Epp J.B., Widlanski T.S. (1999). Facile preparation of nucleoside-5′-carboxylic acids. J. Org. Chem..

[35] Harraz D.M., Davis J.T. (2018). A self-assembled peroxidase from 5′-GMP and heme. Chem. Commun..

[36] Bai J., Sun X., Wang H., Li C., Qiao R. (2020). Guanosine-based self-assembly as an enantioselective catalyst scaffold. J. Org. Chem..

[37] Banwell E.F., Piette B.M., Taormina A., Heddle J.G. (2018). Reciprocal nucleopeptides as the ancestral Darwinian self-replicator. Mol. Biol. Evol..

[38] Van der Gulik P.T., Speijer D. (2015). How amino acids and peptides shaped the RNA world. Life.

[39] Smith J.E., Mowles A.K., Mehta A.K., Lynn D.G. (2014). Looked at life from both sides now. Life.

