# Supplementary material for: Impact of C-Terminal Chemistry on Self-Assembled Morphology of Guanosine Containing Nucleopeptides
Source: Molecules. 2020 Nov 24;25(23):5493. doi: 10.3390/molecules25235493 (PMC7727710; doi:10.3390/molecules25235493)
Supplement: Supplementary file 1 [file molecules-25-05493-s001.pdf]

Supplementary Materials: Impact of C-terminal Chemistry on Self-assembled Morphology of Guanosine Containing Nucleopeptides

Katherine Boback, Katherine Bacchi, Sarah O'Neill, Samantha Brown, Jovelt Dorsainvil and Jillian E. Smith-Carpenter *

Department of Chemistry and Biochemistry, Fairfield University, 1073 N. Benson Rd, Fairfield, CT 06824, USA; kboback21@amherst.edu (K.B.); katherine.bacchi@student.fairfield.edu (K.B.); sarah.oneill@student.fairfield.edu (S.O.); samantha.brown2@student.fairfield.edu (S.B.); jovelt.dorsainvil@student.fairfield.edu (J.D.)

* Correspondence: jsmith-carpenter@fairfield.edu; Tel.: +205-254-4000

Supporting Materials Contents:

^{1}H NMR of 2',3'-O-isopropylideneguanosine-5'-carboxylic acid (Figure S1)………… 2
HPLC and MALDI-TOF of gsGKFF-OH (Figure S2) ……………………………………2
^{1}H NMR of gsGKFF-OH (Figure S3) …………………………………………………….3
HPLC and MALDI-TOF of gsGKFF-NH_2 (Figure S4) ……………………………….…3
^{1}H NMR of gsGKFF-NH_2 (Figure S5) ……………………………………………………4
Vial inversion test (Figure S6) ……………………………...…….…………………………… 4
Second derivative FTIR spectrum (Figure S7) ……………………………………………………5
Nanofiber width distribution (Figure S8) ………………………………………………………… 5

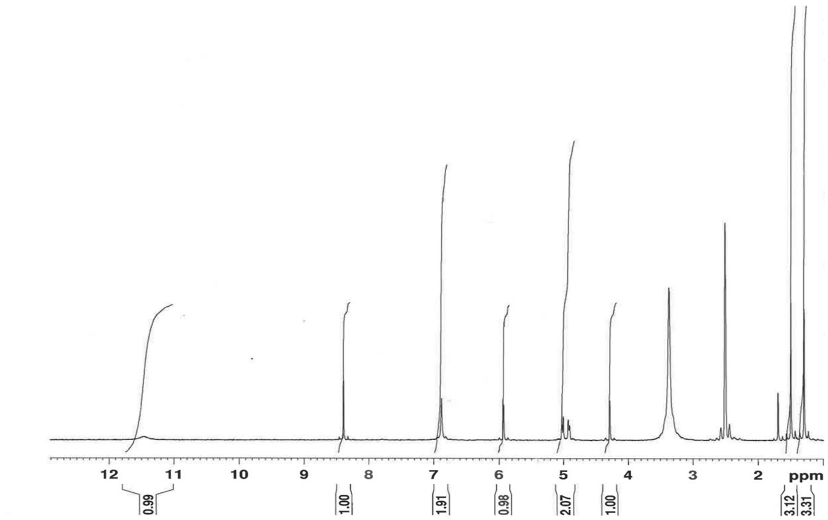

Figure S1: ^{1}H NMR of 2′,3′-*O*-isopropylideneguanosine-5′-carboxylic acid.

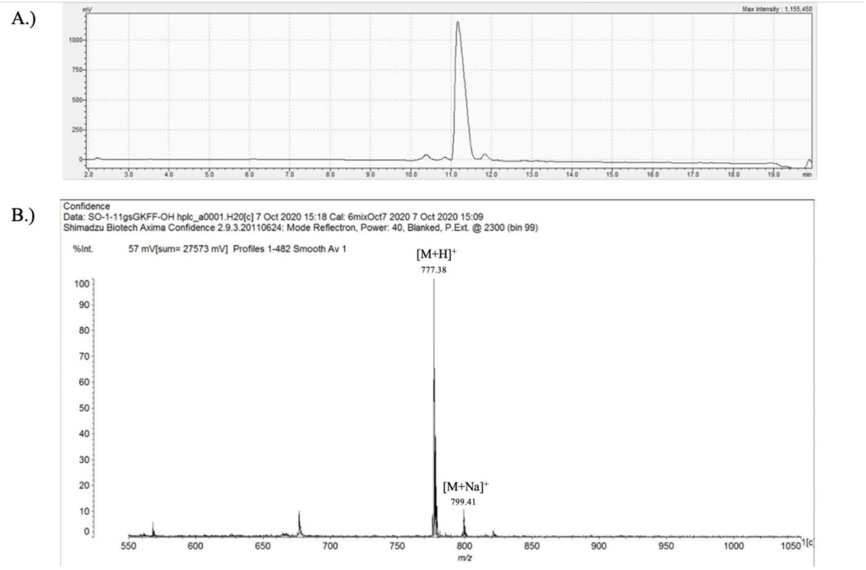

Figure S2. Characterization of purified gsGKFF-OH. **A**) HPLC chromatogram and **B**) MALDI TOF mass spectrum of gsGKFF-OH. Exact Mass: 776.32 g/mol.

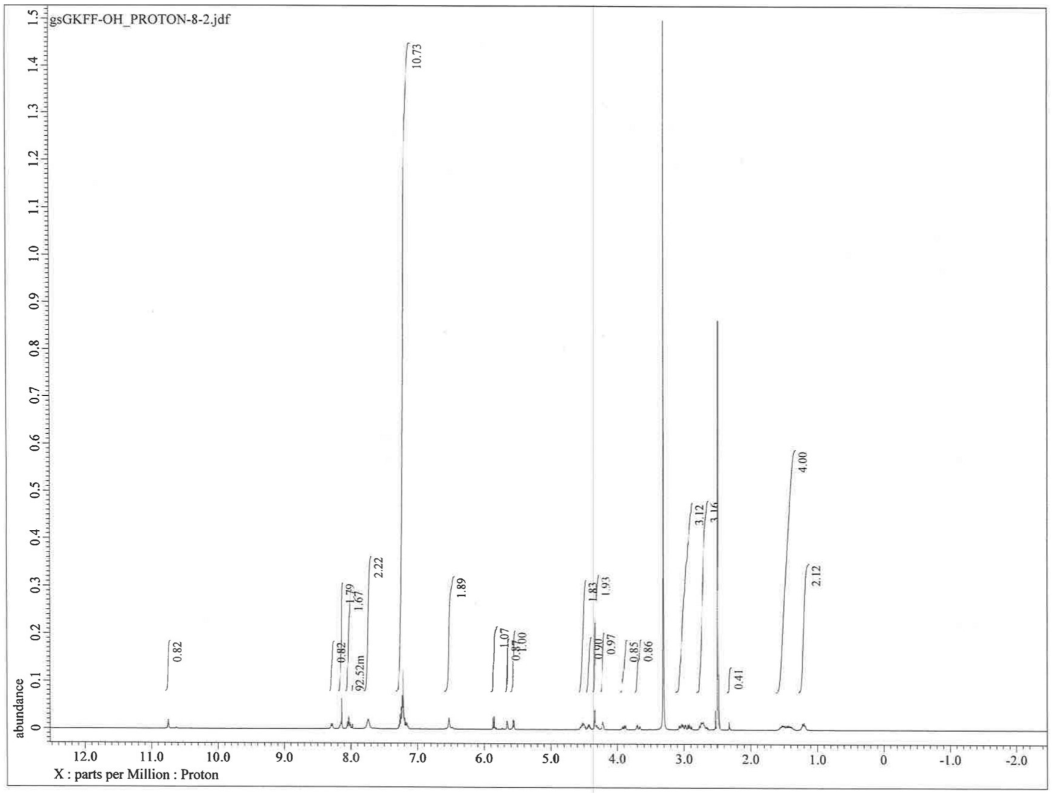

Figure S3. ^{1}H NMR of purified gsGKFF-OH.

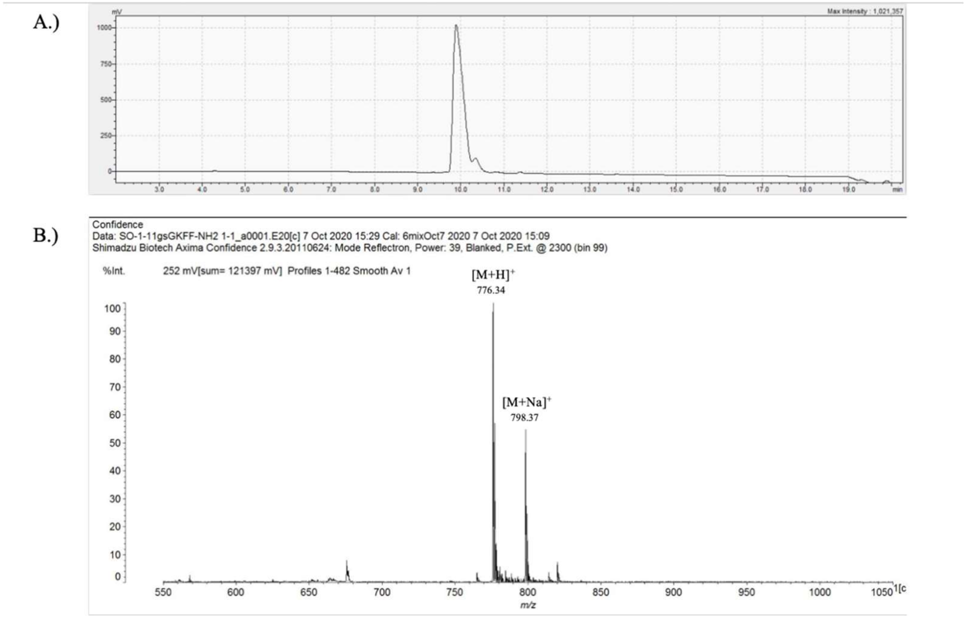

Figure S4. Characterization of purified gsGKFF-NH2. **A**) HPLC chromatogram and **B**) MALDI TOF mass spectrum of gsGKFF-NH2. Exact mass: 775.34 g/mol.

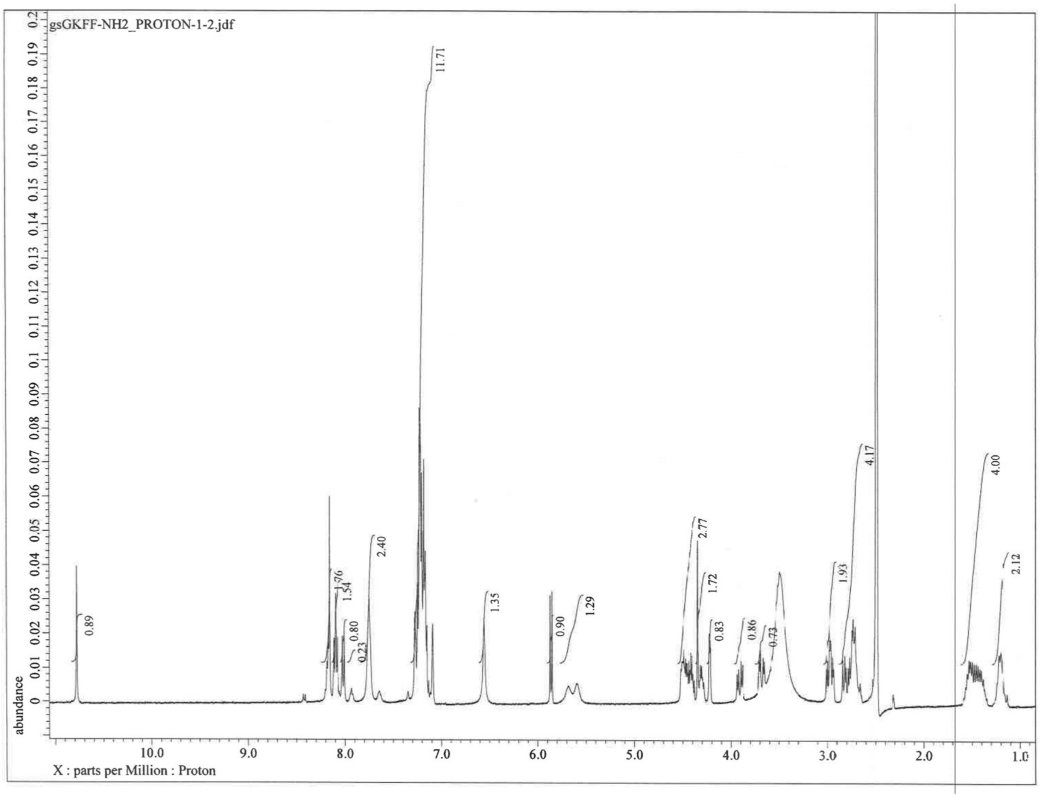

Figure S5. ^{1}H NMR of purified gsGKFF-NH_2.

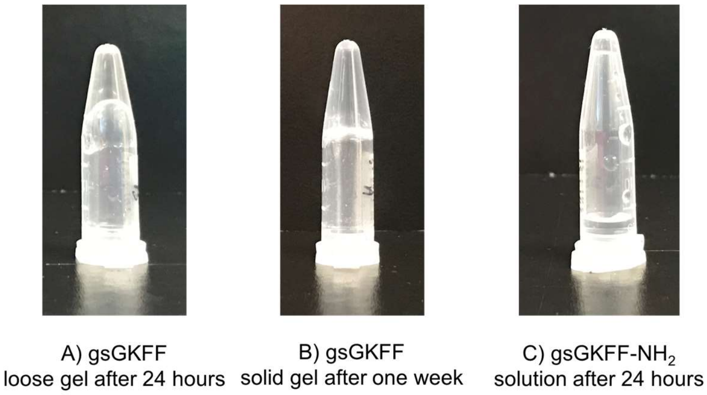

Figure S6. Vial inversion test of nucleopeptides assembled in 20% acetonitrile (*v*/*v*). Nucleopeptide gsGKFF-OH is a loose gel after 24 hours (**A**) but over time stiffens to a transparent gel that holds in place during vial inversion. The hydrogel remains transparent and stable after 1 week (**B**). The assembled gs-GKFF-NH_2 remains soluble and remains a solution after 24 hours (**C**) and longer.

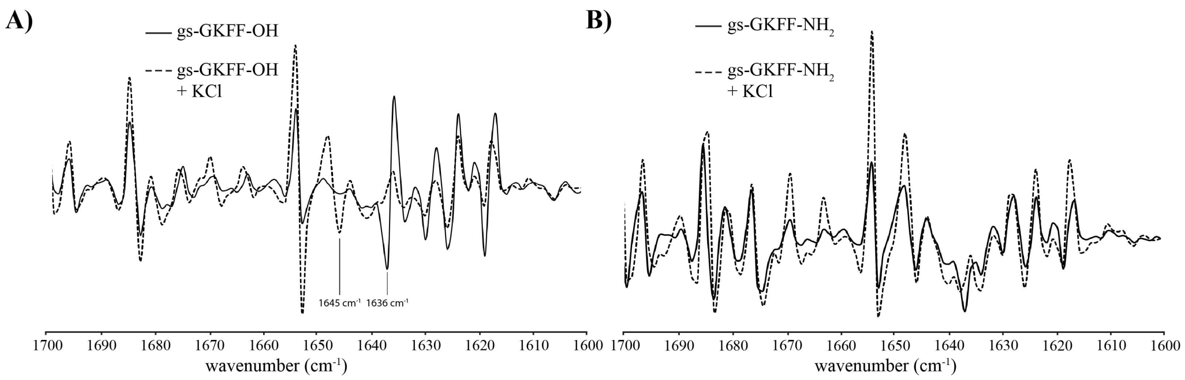

Figure S7. Second derivative FTIR spectra in the absence of KCl (solid line) and presence of 1 eq. KCl (dashed line) for nucleopeptide assemblies of gs-GKFF-OH (**A**) and gs-GKFF-NH_2 (**B**) after one week of assembly.

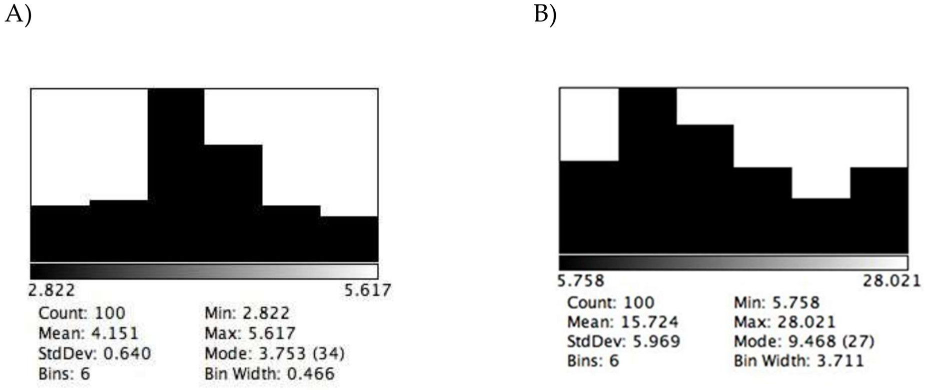

Figure S8. Nanofiber widths measured from TEM of gs-GKFF-OH assembled with 1 eq. KCl in 20% acetonitrile (*v*/*v*). Measurements were taken of both individual striations seen within nanofibers (**A**) and width of nanofibers (**B**). Widths were measured using ImageJ [1].

References

1) C. A. Schneider, W. S. Rasband, K. W. Eliceiri. "NIH Image to ImageJ: 25 Years of image analysis. Nat. Methods **2012**, *9*, 671-675.
